# Effects of adding bile acids to dietary storage japonica brown rice on growth performance, meat quality, and intestinal microbiota of growing–finishing Min pigs

**DOI:** 10.3389/fvets.2024.1349754

**Published:** 2024-04-22

**Authors:** Chuanqi Wang, Kexin Zheng, Dali Wang, Hao Yu, Yun Zhao, Hengtong Fang, Jing Zhang

**Affiliations:** College of Animal Sciences, Jilin University, Changchun, China

**Keywords:** storage japonica brown rice, growth performance, intestinal microbiota, meat quality, Min pigs

## Abstract

**Introduction:**

This study investigated the effects of storage japonica brown rice (SJBR) and bile acids (BA) on the growth performance, meat quality, and intestinal microbiota of growing–finishing Min pigs.

**Methods:**

A total of 24 healthy Min pigs with a similar body weight of 42.25 ± 2.13 kg were randomly divided into three groups with eight replicates of one pig each. The groups were as follows: CON (50% corn), SJBR (25% corn +25% SJBR), and SJBR + BA (25% corn +25% SJBR +0.025% hyodeoxycholic acid). The experimental period lasted from day 90 (the end of the nursery phase) to day 210 (the end of the finishing phase).

**Results:**

The results showed the following: (1) Compared with the CON group, there was no significant difference in the average daily gain (ADG) and average daily feed intake (ADFI) of the SJBR and SJBR + BA groups, and the feed conversion ratio (FCR) was significantly decreased (*p* < 0.05). (2) Compared with the CON group, the total protein (TP) content in the serum was significantly increased, and the blood urea nitrogen (BUN) content was significantly decreased (*p* < 0.05) in the SJBR and SJBR + BA groups; moreover, HDL-C was significantly higher by 35% (*p* < 0.05) in the SJBR + BA group. (3) There were no significant differences in carcass weight, carcass length, pH, drip loss, cooking loss, and shear force among the groups; the eye muscle area was significantly increased in the SJBR group compared with the CON group (*p* < 0.05); back fat thickness was significantly decreased in the SJBR + BA group compared with the SJBR group (*p* < 0.05); and the addition of SJBR significantly increased the mRNA expression of MyHC I in the *longissimus dorsi* (LD) muscle of growing–finishing Min pigs (*p* < 0.05). (4) The cecal bacteria were detected using 16S rDNA, and the proportion of *Lactobacillus* was increased gradually at the genus level, but there was no significant difference among the different groups.

**Conclusion:**

In conclusion, 25% SJBR can improve the growth performance and increase the abundance of intestinal beneficial bacteria, and based on this, adding bile acids can reduce the back fat thickness of growing–finishing Min pigs.

## 1 Introduction

In recent years, with the continued growth of animal production, feed deficiency has become the key constraint affecting modern animal husbandry development in China (1). Additionally, the contradiction between humans and animals competing for food is becoming increasingly prominent, and the development of new feed raw materials has become a research hotspot (2). Corn is the main energy feed ingredient worldwide, and the nutritional value of brown rice is equivalent to that of corn (3). Brown rice is obtained from hulled rice, which has similar effective energy value, essential amino acids, crude protein, mineral, and vitamin content to corn. Thus, it can potentially replace feed corn (4). Brown rice contains various phenolic acids, which have antioxidant activity that protects cells from oxidative damage and is one of the most common antioxidants in the diet (5, 6). Studies have shown that applying germinated brown rice extract to obese mice induced by a high-fat diet significantly reduces serum triglyceride and total cholesterol levels by downregulating genes involved in lipid synthesis, thereby improving lipid distribution in mice (7). Most importantly, studies have reported that partially or completely replacing corn with brown rice can achieve the same feeding effect as corn for livestock and poultry (8, 9).

Compared to fresh brown rice, storage brown rice stored for 3 years has almost no difference in most nutritional parameters. Nevertheless, the decomposition of crude fat in storage brown rice produces a large amount of free fatty acids, which can oxidize and produce an unpleasant odor (10). In addition, a prolonged storage time can significantly reduce the activities of rice amylase, peroxidase, and polyphenol oxidase (11). Storage brown rice can be used as a high-quality energy raw material to replace corn with proper processing to eliminate the presence of fungal toxins and anti-nutritional factors (12).

Bile acids are biotransformed in the intestine mainly by anaerobic interactions of *Bacteroides*, *Eubacterium*, *Clostridium*, and *Lactobacillus* and are restored to free bile acids after binding to taurine and glycine via bile salt hydrolase-catalyzed uncoupling of conjugated bile acids (13). Bile acids reduce the production of short-chain fatty acids (SCFA), a metabolite of the intestinal microbiota, and have bacteriostatic activity, effectively inhibiting the growth and proliferation of intestinal pathogenic bacteria, maintaining the balance of intestinal micro-ecology, protecting the intestinal mucosa from bacterial invasion, and regulating the immune function of the body (14, 15). Bile acids promote the digestion and absorption of fats while stimulating bile secretion, helping to improve immunity and regulate the gut microbiota (16, 17). In addition to regulating glycolipid metabolism, bile acids also regulate intestinal cell proliferation and resist intestinal oxidative damage (18). After implanting a catheter in the duodenum, enteral malnourished piglets were treated with 30 mg/kg body weight of chenodeoxycholic acid, which could significantly increase their intestinal mass and ileal villus height/eye socket depth ratio and promote their intestinal development (19). Therefore, using storage brown rice as an energy feed substitute for corn and improving its utilization rate through additives may alleviate various problems such as corn supply–demand contradiction and pressure to reduce rice inventory. This study was devoted to investigating the feasibility of partially replacing corn with storage brown rice and the effect of bile acids as additives in improving the performance of Min pigs.

Min pig, one of the excellent local pig breeds in China, has cold resistance, rough feeding resistance, strong disease resistance, and good meat quality (20). Although there have been studies on the application of dietary brown rice in poultry and swine, the extent to which brown rice replacing part corn affects the meat quality and gut microbiota distribution of Chinese local pig breeders is still unclear. This study assumes that storage brown rice is a good substitute for corn and does not affect the normal growth of Min pigs during the growing–finishing period. Therefore, this study aimed to investigate how replacing some corn in the diet with storage brown rice and adding bile acids affected the growth performance, carcass traits, and meat quality of growing–finishing Min pigs.

## 2 Materials and methods

### 2.1 Experimental design and animals

The bile acid (hyodeoxycholic acid, purity >30%) used in this study was obtained from Shandong Longchang Animal Health Products Co., Ltd. (Jinan, China). A total of 24 healthy 83-day-old Min pigs (half male and half female) with an average body weight of 42.25 ± 2.13 kg were randomly allotted to three experimental groups with eight duplicates and one pig per replicate. The dietary treatments included the following: (1) CON (50% corn), (2) SJBR (25% corn +25% storage japonica brown rice), and (3) SJBR + BA (25% corn +25% storage japonica brown rice +0.025% bile acids). Each treatment had eight replicates, with one pig per replicate. The Min pigs were fed *ad libitum* and had free access to water with a temperature of 16–22°C and a relative humidity of 60–70% throughout the trial. The pigs were adaptively reared for 7 days. The experimental period lasted from day 90 (the end of the nursery phase) to day 210 (the end of the finishing phase). The experimental diet was formulated based on the Nutrient Requirements of Swine in China (GB/T 39235–2020) for growing–finishing pigs (21). The compositions and nutrition levels of the experimental diets are listed in Table 1. The initial body weight and final body weight were measured on the first and last days of the formal experimental period, and the daily feed intake was recorded to calculate ADG, ADFI, and FCR.

**Table 1:** The composition and nutrition levels of experimental diets (as-fed basis).

Items	Treatment^1^		
Ingredients (%)	CON	SJBR	SJBR + BA
Corn	50	25	25
Storage japonica brown rice	0	25	25
Peanut meal	2	2	2
DDGS	9	9	9
Rice bran	10	10	10
Wheat middling	12	12	12
Rice bran meal	13	13	13
Limestone	1.25	1.25	1.25
CaHPO_4_	0.25	0.25	0.25
NaCl	0.4	0.4	0.4
Phytase	0.02	0.02	0.02
Lysine	0.34	0.34	0.34
Threonine	0.07	0.07	0.07
Methionine	0.03	0.03	0.03
Tryptophan	0.03	0.03	0.03
Lysine residue	0.8	0.8	0.8
t-BHQ	0.01	0.01	0.01
Bile acids	—	—	0.025
Zeolite powder	0.07	0.07	0.07
Choline bitartrate	0.23	0.23	0.205
Premix^2^	0.5	0.5	0.5
Total	100	100	100
Nutrition levels (%)^3^			
Metabolic energy (MJ/kg)	12.16	12.20	12.20
Crude protein	13.30	13.30	13.30
Ether extract	4.49	4.12	4.12
Crude fiber	4.44	3.91	3.91
Ash	4.75	4.84	4.84
Ca	0.58	0.58	0.58

^1^CON, control group; SJBR, storage japonica brown rice group; SJBR + BA, storage japonica brown rice + bile acid group.

^2^The premix supplied the following (per kg diet): 20 mg cupric carbonate, 0.3 mg potassium iodate, 80 mg ferric citrate, 20 mg manganese carbonate, 0.3 mg sodium selenate, 70 mg zinc carbonate, 5,000 IU vitamin A, 4,500 IU vitamin D3, 90 mg vitamin E, 8 mg vitamin K3, 4 mg vitamin B1, 30 μg vitamin B12, 0.4 mg biotin, 0.6 mg folic acid, 14 mg pantothenic acid, 30 mg nicotinic acid.

^3^Metabolic energy was calculated value, and other nutrient levels were measured values.

### 2.2 Sample collection

At the end of the feeding experiment, all the pigs in each treatment were transported to a modern slaughterhouse and slaughtered for sample collection. After arriving at the slaughterhouse, all pigs were allowed to rest for 4 h. Then, the jugular vein blood of selected pigs was bled, which complies with the current regulations applicable to the slaughterhouse. The carcasses were scalded, eviscerated, and vertically separated along the midline. The carcass weight was recorded to calculate the slaughter rate. The back fat thickness was measured by a three-point method: the back fat depth at the left carcass of the first rib, the last rib, and the last lumbar spine. The right carcass at the 10th rib was used for the loin-eye area (height × width/0.7 cm^2^), meat color, shearing force, drip loss, and cooking loss determination. The *longissimus dorsi* (LD) muscle samples (the 10th rib of the right carcass) were separated and frozen at −80°C until further analysis.

### 2.3 Chemical analysis of plasma

The blood samples (10 mL) from each pig were collected using heparin tubes and centrifuged at 4,000 rpm for 10 min after slaughter immediately. Then, the plasma samples were separated and stored in 1.5 mL Eppendorf tubes frozen at −20°C until further analysis. The concentrations of total protein (TP, Cat# A045-4-2), alkaline phosphatase (ALP, Cat# A059-2-2), albumin (ALB, Cat# A028-2-1), glutamic-pyruvic transaminase (ALT, Cat# C009-2-1), glutamic-oxalacetic transaminase (AST, Cat# C010-2-1), blood urea nitrogen (BUN, Cat# C013-2-1), creatinine (CREA, Cat# C011-2-1), uric acid (UA, Cat# C012-2-1), total cholesterol (T-CHO, Cat# A111-1-1), high-density lipoprotein cholesterol (HDL-C, Cat# A112-1-1), low-density lipoprotein cholesterol (LDL-C, Cat# A113-1-1), and triglycerides (TG, Cat# A110-1-1) were measured by a Unicel DxC 800 Synchron (Clinical System, Beckman Coulter, Fullerton, CA, United States), following the kit instructions from the Nanjing Jiancheng Bioengineering Institute (Nanjing, China).

### 2.4 Meat quality measurements

The LD muscle samples (the 10th rib of the right carcass) were selected for meat quality measurement. The meat color was measured at 45 min and 24 h after slaughter with a hand-held colorimeter (CR-410, Konica Minolta Sensing Inc., Osaka, Japan), respectively. Similarly, at 45 min and 24 h after slaughter, the pH probe (Matthäus pH Star, Germany) was inserted into the LD muscle to measure the pH value. The LD muscle samples were taken, and the initial weight was recorded. Then, the sample was hung and placed in a sealed fresh-keeping box, suspended at 4°C for 24 h. Then, it was reweighed to calculate the dripping loss. The meat samples were boiled in the bag with 75°C water in a water bath until the internal temperature of the meat reached 70°C, and then dried and cooled at room temperature. Cooking loss was determined by calculating the weight loss during cooking.

### 2.5 Quantitative real-time PCR for target genes

Total RNA from the LD muscle and intestinal tissue was isolated using TRIzol reagent (Thermo Fisher Scientific Co., MA, USA). The concentration and purity of total RNA were measured by a nanophotometer (Thermo Fisher Scientific Co., MA, USA), followed by measuring the absorbance at 260/280 nm. Then, the extracted RNA (1,000 ng) was reverse-transcribed into cDNA using the Bio-DL Life ECO Gradient Qualitative PCR Gene Amplifier (Hangzhou, China). Subsequently, the cDNA was used as a template for RT-qPCR using a reverse transcription reagent kit (TransGen Biotech, Beijing, China) through an ABI PRISM 7500 SDS thermal cycler apparatus (Applied Biosystems, Foster City, CA, United States). The primer sequences (Table 2) were designed through NCBI and synthesized by Sangon Biotech (Shanghai, China). The expression levels of mRNA related to myosin heavy chain of muscle fiber and intestinal tight junction protein were calculated by the 2^−△△Ct^ method as described in the previous study (22). The expression level of GAPDH was determined as a reference gene, and the transcription levels of target genes were normalized to GAPDH mRNA in each sample.

**Table 2:** Primer sequences and the PCR product-amplified fragments used in RT-qPCR.

Genes	Accession no.	Product size (bp)	Sequences (5′ → 3′)
MyHC I	NM_213855.2	133	F: AGTGCAGGCGGAACAAGACAATC
			R: AGCATTCATCTCCTCCTCGTCCTC
MyHC IIx	NM_001104951.2	127	F: ACTGAGGAAGACCGCAAGAACATTC
			R: ACTTGGAGAGGTTGACGTTGGATTG
Myoglobin	NM_214236.1	115	F: CTCATCAGGCTCTTTAAGGGTCACC
			R: TGTTGCCGTGCTTCTTCAGGTC
MyHC IIa	NM_214136.1	153	F: ACAGTGAAGACGGAAGCAGG
			R: TGCGTAACGCTCTTTGAGGT
GAPDH	NM_001206359.1	104	F: ATCCTGGGCTACACTGAGGAC
			R: AAGTGGTCGTTGAGGGCAATG

MyHC I, myosin heavy chain I; MyHC IIx, myosin heavy chain IIx; MyHC IIa, myosin heavy chain IIa.

### 2.6 The 16S sequencing and data analyses

The total DNA from cecal bacteria was extracted according to the manufacturer’s instructions. (QIAGEN QIAamp PowerFecal DNA Kit, Germany). The total DNA was eluted in 50 μL of elution buffer and stored at −80°C until measurement in the PCR. Universal primers 341F and 805R were used for PCR amplification of the V3–V4 hypervariable regions of 16S rDNA genes (341F, 5′-CCTACGGGNGGCWGCAG-3′; 805R, 5′-GACTACHVGGGTATCTAATCC-3′). The 5′ ends of the primers were tagged with specific barcodes per sample and sequenced with universal primers. The PCR conditions to amplify the prokaryotic 16S fragments consisted of an initial denaturation at 98°C for 30 s, 32 cycles of denaturation at 98°C for 10 s, annealing at 54°C for 30 s, extension at 72°C for 45 s, and then final extension at 72°C for 10 min. The PCR products were confirmed with 2% agarose gel electrophoresis. Throughout the DNA extraction process, ultrapure water, instead of a sample solution, was used as a negative control to exclude the possibility of false-positive PCR results. The PCR products were purified by AMPure XP Beads (Beckman Coulter Genomics, Danvers, MA, United States) and quantified by Qubit (Invitrogen, USA). The amplicon pools were prepared for sequencing, and the size and quantity of the amplicon library were assessed on Agilent 2100 Bioanalyzer (Agilent, United States) and with the Library Quantification Kit for Illumina (Kapa Biosciences, Woburn, MA, United States), respectively.

Samples were sequenced on an Illumina NovaSeq platform according to the manufacturer’s recommendations provided by LC-Bio (Hangzhou, China). Quality filtering on the raw reads was performed under specific filtering conditions to obtain high-quality clean tags according to fqtrim (v0.94). Chimeric sequences were filtered using VSEARCH software (v2.3.4). Alpha diversity was applied in analyzing the complexity of species diversity for a sample through five indices, namely, Chao1, observed species, goods coverage, Shannon, and Simpson, and all the indices in our samples were calculated using QIIME2. Beta diversity was calculated by QIIME2; the graphs were plotted using the R package. BLAST was used for sequence alignment, and the feature sequences were annotated with the SILVA database for each representative sequence.

### 2.7 Statistical analysis

The experimental data were analyzed using SPSS 22.0 (SPSS Inc., Chicago, IL, United States) with a one-way ANOVA model, followed by Duncan’s multiple range tests. The data in figures and tables were shown as the mean ± standard error of means (SEM). In this study, differences were shown to be significant at *p* < 0.05, and trends toward significance were at 0.05 < *p* < 0.10.

## 3 Results

### 3.1 Growth performance

The growth performance of growing–finishing Min pigs with different treatments is shown in Table 3. In this study, the SJBR and BA supplementation significantly reduced the ratio of ADFI-to-ADG (F/G) compared to the CON treatment (*p* < 0.05). Concurrently, the average daily gain (ADG) and average daily feed intake (ADFI) were not affected by the dietary replacement of corn with SJBR and bile acids.

**Table 3:** Effects of adding bile acids to dietary storage japonica brown rice on the growth performance of growing–finishing Min pigs.

Items	Treatments			*p*-value
	CON	SJBR	SJBR + BA	
IBW, kg	41.12 ± 2.31	41.81 ± 1.96	40.71 ± 2.91	0.95
FBW, kg	102.13 ± 6.16	99.31 ± 5.83	94.00 ± 7.06	0.58
ADG, kg	0.38 ± 0.03	0.43 ± 0.02	0.43 ± 0.06	0.67
ADFI, kg	2.25 ± 0.13	2.29 ± 0.15	2.22 ± 0.24	0.96
FCR	5.97 ± 0.15^a^	5.38 ± 0.13^b^	5.27 ± 0.19^b^	0.02

In the same row, values with no letter or the same small letter superscripts mean no significant difference (*p* > 0.05), whereas values with different small letter superscripts mean significant differences (*p* < 0.05).

IBW, initial body weight; FBW, finial body weight; ADG, average daily gain; ADFI, average daily feed intake; FCR, feed conversion ratio = ADFI (kg)/ADG (kg).

CON, control group; SJBR, storage japonica brown rice group; SJBR + BA, storage japonica brown rice + bile acid group. Data are expressed as the mean ± SEM (*n* = 8).

### 3.2 Plasma biochemical index

As shown in Table 4, there is no significant difference in the contents of alanine aminotransferase, aspartate aminotransferase, alkaline phosphatase, albumin, uric acid, total cholesterol, triglyceride, and low-density lipoprotein cholesterol between groups. Compared with the CON group, the total protein content in the SJBR group and SJBR + BA group significantly increased (*p* < 0.05), while the urea nitrogen content significantly decreased (*p* < 0.05). Meanwhile, the content of high-density lipoprotein cholesterol in the SJBR + BA group was significantly increased by 35% (*p* < 0.05).

**Table 4:** The effects of adding bile acids to dietary storage japonica brown rice on the plasma biochemical indices of growing–finishing Min pigs.

Items	Treatments			*p*-value
	CON	SJBR	SJBR + BA	
ALT, U/L	55.30 ± 3.53	54.13 ± 2.21	51.25 ± 2.06	0.56
AST, U/L	65.80 ± 2.19	62.53 ± 2.71	59.88 ± 5.03	0.51
ALP, U/L	6.30 ± 0.67	5.95 ± 0.66	5.72 ± 0.61	0.82
TP, g/L	83.28 ± 0.78^b^	91.07 ± 1.03^a^	88.23 ± 1.92^a^	0.003
ALB, g/L	38.02 ± 0.80	38.73 ± 1.55	41.08 ± 0.76	0.16
BUN, mmol/L	5.96 ± 0.20^a^	5.00 ± 0.20^b^	4.79 ± 0.33^b^	0.01
UA, μmol/L	15.75 ± 0.42	16.65 ± 0.32	16.30 ± 0.29	0.21
TCHO, mmol/L	1.98 ± 0.12	1.98 ± 0.04	2.08 ± 0.04	0.61
TG, mmol/L	0.37 ± 0.04	0.35 ± 0.03	0.32 ± 0.05	0.63
HDL-C, mmol/L	0.40 ± 0.03^b^	0.46 ± 0.02^ab^	0.54 ± 0.03^a^	0.02
LDL-C, mmol/L	0.75 ± 0.07	0.65 ± 0.03	0.60 ± 0.06	0.14

ALT, glutamic-pyruvic transaminase; AST, glutamic-oxalacetic transaminase; ALP, alkaline phosphatase; TP, total protein; ALB, albumin; CREA, creatinine; BUN, blood urea nitrogen; UA, uric acid; TG, triglyceride; T-CH, total cholesterol; HDL-C, high-density lipoprotein cholesterol; LDL-C, low-density lipoprotein cholesterol.

In the same row, values with no letter or the same small letter superscripts mean no significant difference (*p* > 0.05), whereas values with different small letter superscripts mean significant differences (*p* < 0.05).

CON, control group; SJBR, storage japonica brown rice group; SJBR + BA, storage japonica brown rice + bile acid group. Data are expressed as the mean ± SEM (*n* = 8).

### 3.3 Carcass traits and meat quality

**Figure 1:**
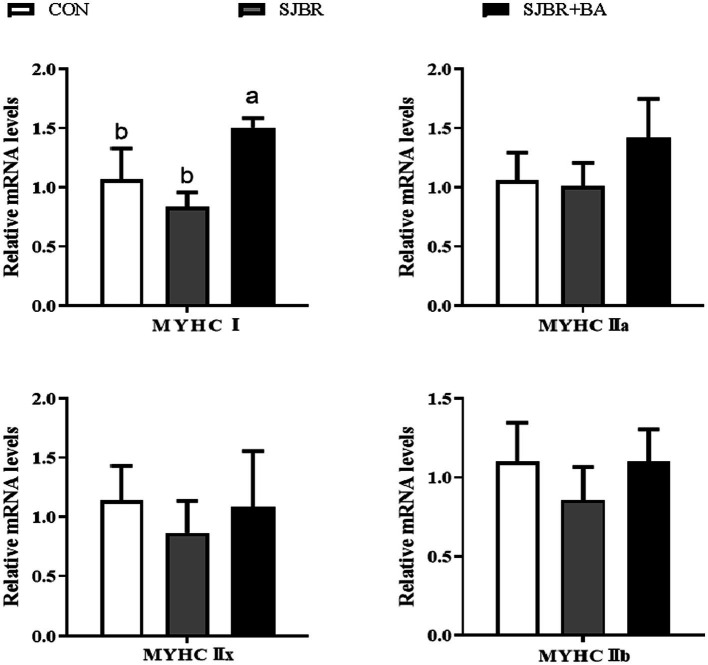
The effects of adding bile acids to dietary storage japonica brown rice on mRNA expression of myosin heavy chain (MyHC) isoforms in the longissimus dorsi (LD) muscle of growing–finishing Min pigs. CON, control group; SJBR, storage japonica brown rice group; SJBR + BA, storage japonica brown rice + bile acid group. Data in the column chart were expressed as the mean ± SEM (*n* = 6). ^a,b^Bars with different letters were declared significant at *p* < 0.05.

As shown in Table 5, compared with the CON group, the eye muscle area of the SJBR group significantly increased (*p* < 0.05). In addition, the SJBR + BA group significantly reduced back fat thickness compared with the SJBR group (*p* < 0.05). However, there were no significant differences in carcass weight, carcass length, pH _45 min_, pH _24 h_, drip loss, cooking loss, or shear force among the groups. We detected the relative mRNA expression levels of myosin heavy chain (MyHC) subtypes (I, IIa, IIx, and IIb) of four different muscle fiber types (slow oxidative type—I, fast oxidative type—IIa, intermediate type—IIx, and fast glycolytic type—IIb) in the LD muscle. The results showed that the expression level of the MyHC I gene in the LD muscle fibers of the SJBR + BA group was significantly increased (*p* < 0.05), and there was no significant difference in the expression levels of MyHC IIa, MyHC IIx, and MyHC IIb genes between the groups (Figure 1).

**Table 5:** The effects of adding bile acids to dietary storage japonica brown rice on the carcass traits and meat quality of growing–finishing Min pigs.

Items	Treatments			*p*-value
	CON	SJBR	SJBR + BA	
Carcass weight, kg	64.70 ± 1.57	67.10 ± 1.14	67.17 ± 1.59	0.41
Length of carcass, cm	71.90 ± 0.96	71.33 ± 0.92	75.87 ± 1.28	0.11
Eye muscle area, cm^2^	29.24 ± 0.90^b^	32.77 ± 1.01^a^	30.79 ± 0.57^ab^	0.03
Backfat thickness, mm	50.74 ± 0.50^a^	49.87 ± 0.86^a^	46.25 ± 1.31^b^	0.01
pH_45 min_	6.52 ± 0.11	6.48 ± 0.17	6.57 ± 0.15	0.92
pH_24 h_	5.70 ± 0.19	5.74 ± 0.15	5.73 ± 0.19	0.99
Drip loss	3.37 ± 0.07	3.31 ± 0.10	3.28 ± 0.11	0.81
Cooking loss	22.61 ± 0.52	22.17 ± 0.63	22.98 ± 0.49	0.59
shear force, N	49.40 ± 0.99	50.45 ± 1.18	49.31 ± 1.18	0.73

In the same row, values with no letter or the same small letter superscripts mean no significant difference (*p* > 0.05), whereas values with different small letter superscripts mean a significant difference (*p* < 0.05).

CON, control group; SJBR, storage japonica brown rice group; SJBR + BA, storage japonica brown rice + bile acid group. Data are expressed as the mean ± SEM (*n* = 8).

### 3.4 Fecal bacterial community structure

**Figure 2:**
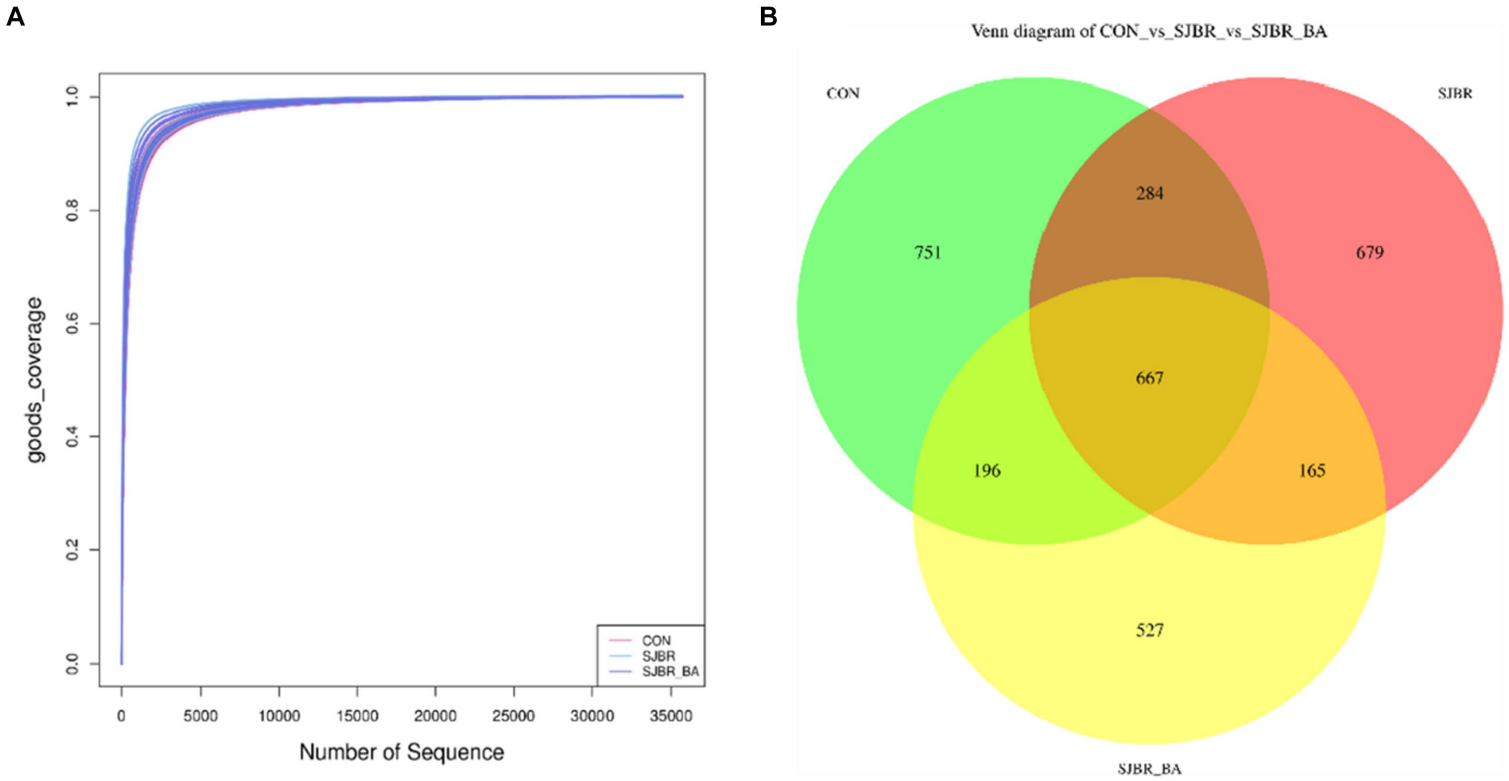
**(A)** Rarefaction curve of three dietary treatments. **(B)** ASV Venn of three dietary treatments. CON, control group; SJBR, storage japonica brown rice group; SJBR + BA, storage japonica brown rice + bile acid group.

To evaluate the impact of SJBR diets on the microbial composition of feces, a total of 1,232,084 V3–V4 16S rRNA effective sequences from the 18 samples, with an average of 68,449 sequences per sample, were used for subsequent analysis (Table 6). The goods coverage showed that the sampling in each group provided sufficient OTU coverage (Figure 2A). Overall, 1,898, 1,795, and 1,555 ASVs were recorded in the CON, SJBR, and SJBR + BA groups, respectively; of which, 667 OTUs were shared among the three groups (Figure 2B).

**Table 6:** Valid data statistics table.

Sample	Raw_Tags	Raw_Bases	Valid_Tags	Valid_Bases	Valid%	Q20%	Q30%	GC%
CON1	85,977	42.99 M	69,627	28.67 M	80.98	95.99	89.54	52.78
CON2	87,604	43.80 M	71,808	29.45 M	81.97	96.56	90.99	53.02
CON3	84,821	42.41 M	68,494	28.29 M	80.75	96.42	90.73	52.70
CON4	82,855	41.43 M	67,559	27.87 M	81.54	96.54	90.98	52.66
CON5	84,226	42.11 M	70,694	29.17 M	83.93	96.41	90.64	52.65
CON6	84,988	42.49 M	69,508	28.67 M	81.79	95.76	88.92	52.79
SJBR_2	80,570	40.28 M	65,759	27.22 M	81.62	96.78	91.50	52.92
SJBR_3	86,097	43.05 M	70,832	29.37 M	82.27	96.40	90.68	52.59
SJBR_4	85,839	42.92 M	65,138	26.97 M	75.88	91.99	81.31	53.15
SJBR_5	81,354	40.68 M	65,738	27.10 M	80.80	96.74	91.38	52.80
SJBR_6	83,744	41.87 M	67,734	28.39 M	80.88	96.44	90.75	51.79
SJBR_1	80,545	40.27 M	65,962	27.33 M	81.89	96.63	91.19	52.99
SJBR_BA_1	82,311	41.16 M	69,187	28.89 M	84.06	96.60	91.08	52.13
SJBR_BA_2	81,378	40.69 M	65,507	27.13 M	80.50	94.45	86.15	53.16
SJBR_BA_3	87,173	43.59 M	68,647	28.44 M	78.75	96.69	91.23	52.72
SJBR_BA_4	86,440	43.22 M	71,108	29.56 M	82.26	96.62	91.17	52.22
SJBR_BA_5	87,103	43.55 M	70,683	29.25 M	81.15	94.08	85.56	52.90
SJBR_BA_6	81,445	40.72 M	68,099	28.48 M	83.61	95.50	88.43	52.34

CON, control group; SJBR, storage japonica brown rice group; SJBR + BA, storage japonica brown rice + bile acid group.

### 3.5 Alpha diversity of the fecal microbiome

**Figure 3:**
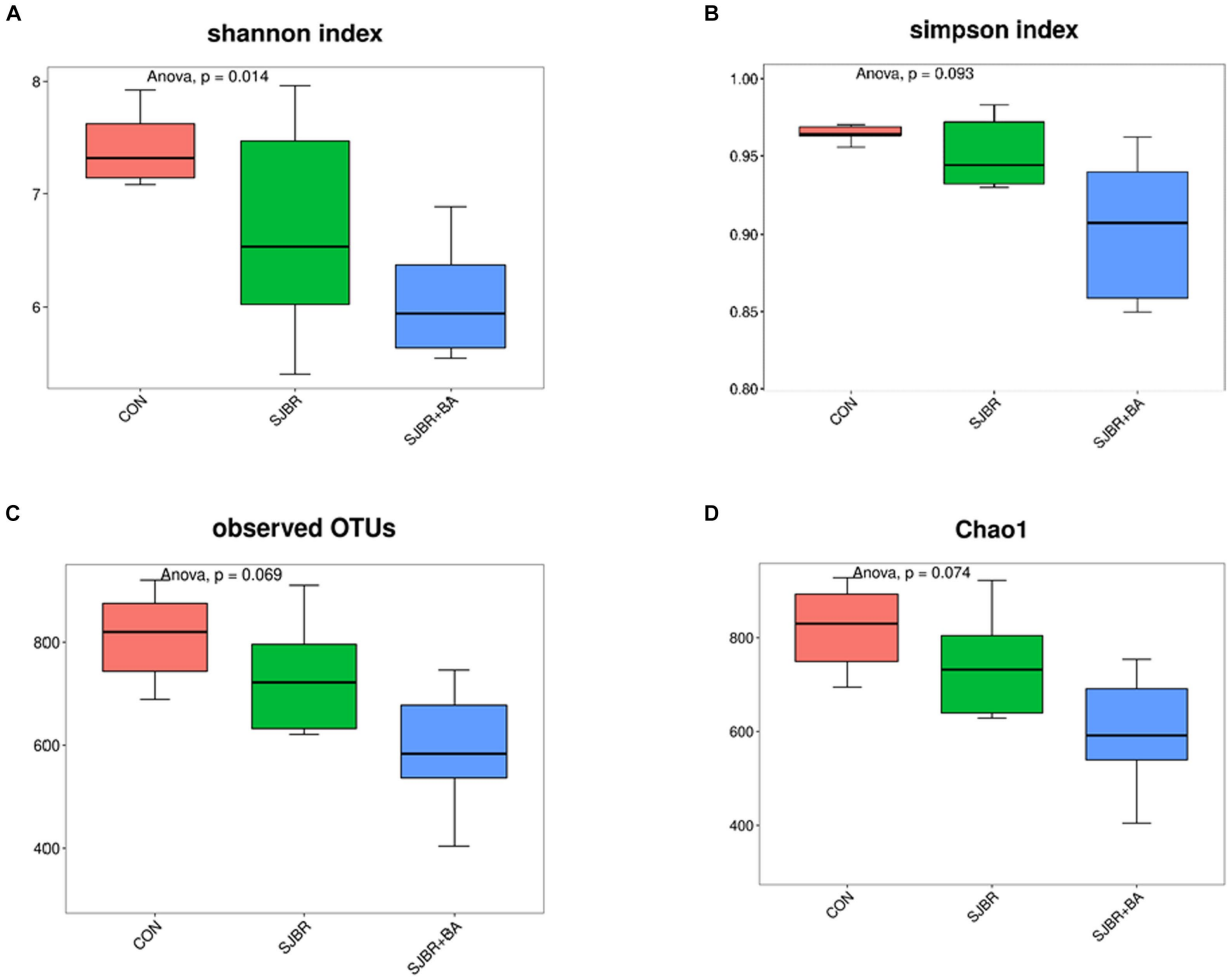
Measurements of fecal microbiome alpha and beta diversity at the amplicon sequence variant (ASV) level. Measurement of alpha diversity at the ASV level using the **(A)** Shannon, **(B)** Simpson, **(C)** observed OTUs, and **(D)** Chao1 among three dietary treatments. Data are expressed as the mean ± SEM (*n* = 6). CON, control group; SJBR, storage japonica brown rice group; SJBR + BA, storage japonica brown rice + bile acid group.

**Figure 4:**
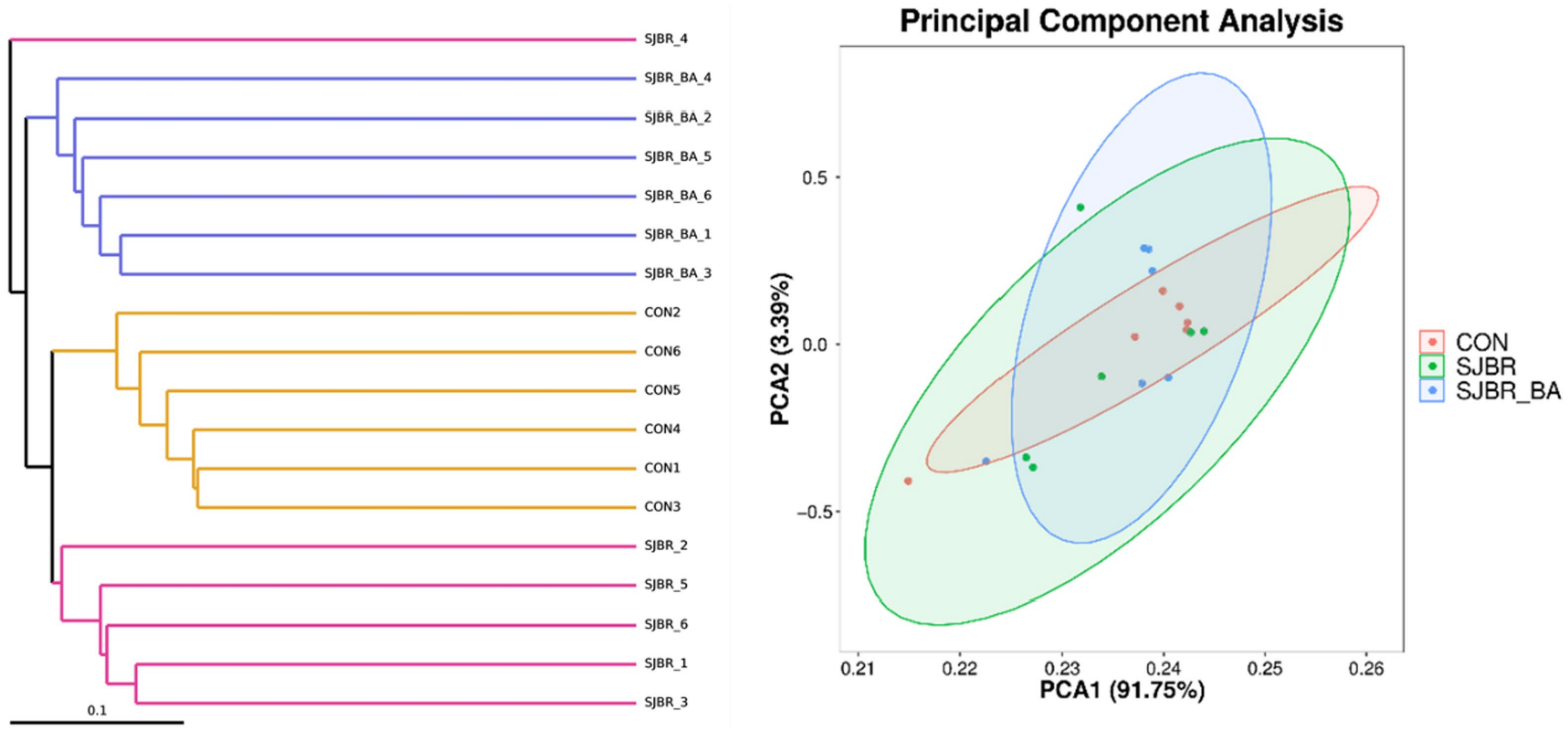
Principal component analysis (PCA) based on the Bray–Curtis distance of all the samples among the three dietary treatments. Data are expressed as the mean ± SEM (*n* = 6). CON, control group; SJBR, storage japonica brown rice group; SJBR + BA, storage japonica brown rice + bile acid group.

To analyze the abundance and diversity of intestinal microbiota, the alpha diversity of samples, including the Chao1 index, observed OTUs, Shannon index, and Simpson index, was performed. As shown in Figure 3, there was no significant difference in the Principal Component Analysis (PCA), Chao1 index, observed OTUs, or Simpson index among different treatment groups, while the Shannon index of the SJBR group significantly decreased (*p* < 0.05) (Figure 4).

### 3.6 Relative abundance of species structure of fecal microbiota

**Figure 5:**
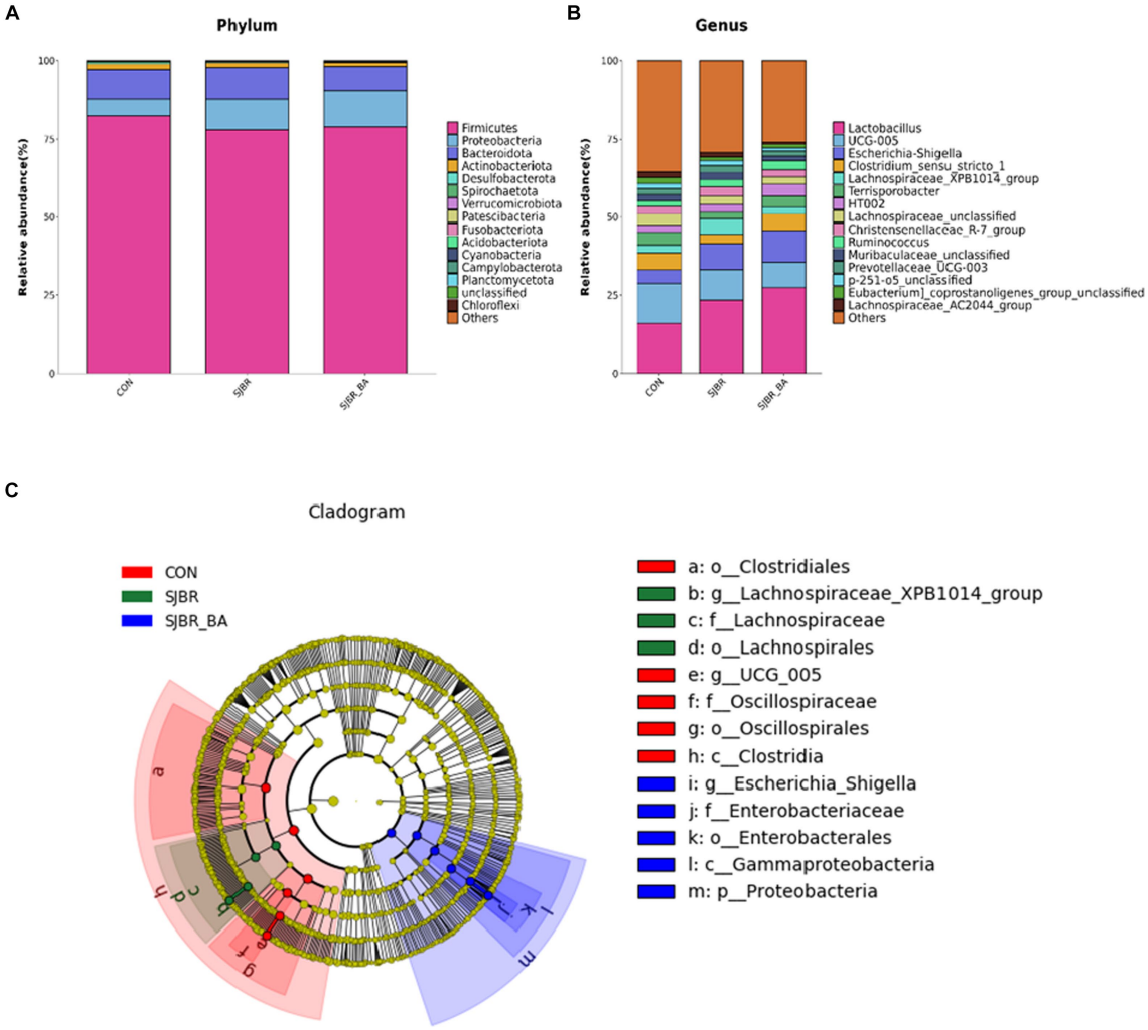
The effects of SJBR and BA on the composition of fecal microbiota from phylum to genus in growing–finishing Min pigs (*n* = 6). Comparison of the major microbes at the **(A)** phylum and **(B)** genus levels among three dietary treatments, respectively. The abundances of the top 15 microbes are expressed as proportions. **(C)** Linear discriminant analysis effect size (LEfSe) analysis based on amplicon sequence variants (ASVs) characterized the microbiomes among three treatments of growing–finishing Min pigs.

At the phylum and genus levels, the relative abundance of species structure in the fecal microbiota in each group of pigs was analyzed. As shown in Figure 5A, *Firmicutes*, *Proteobacteria*, *Bacteroidetes*, and *Actinobacteriota* were predominant in the three dietary treatments at the phylum level. In comparison to CON, the profiling of microbial phyla in SJBR + BA was characterized by a high proportion of *Proteobacteria* (11.83% vs. 5.44%) and a low proportion of *Bacteroidetes* (7.26 vs. 9.09%), and the *Firmicutes* to *Bacteroidetes* ratio was higher in the SJBR + BA group than the SJBR and CON groups. At the genus level (Figure 5B), the proportion of *Lactobacillus* gradually increased (CON 15.93%, SJBR 23.44%, and SJBR + BA 27.42%), with no significant difference. In order to conduct a more detailed study of microorganisms with significant differences between different varieties, we selected the top 15 at the genus level, which showed significant differences in the Kruskal–Wallis test.

## 4 Discussion

The growth performance directly affects the meat production capacity of growing–finishing pigs and the economic benefits of animal husbandry enterprises. The results of this study showed that, compared with the CON group, there were no significant differences in the ADG and ADFI of the SJBR and SJBR + BA groups after replacing half of the corn with storage japonica brown rice, indicating that there is no potential harm to the growth performance of Min pigs. Similar to the current results, there were no significant differences in ADG, ADFI, and FCR between the brown rice replacement group (50, 75, and 100%), and the control group was fed a corn–soybean meal basal diet (3). Zhang et al. also found that the ADG of experimental pigs fed brown rice increased slightly, and the ADFI-to-ADG ratio was reduced, even though there was no significant difference between the brown rice group and the corn group. However, it had a good effect on cost reduction and efficiency in animal production (23). Nevertheless, studies have shown that feeding weaned piglets with brown rice as a 50% substitute for corn resulted in an increase of 2.43 and 2.88% in apparent ileal digestibility of dry matter and energy compared to the control group, respectively (24). The reasons for the different results may be attributed to the different growth stages of the experimental animals selected by researchers, as well as the differences in the storage time of brown rice. Additionally, the substitution of brown rice for corn in swine diets at all feeding stages did not affect or even improve growth performance, most likely due to the slightly higher nutritional value of brown rice compared to corn and its low content of anti-nutritional factors.

There is a certain degree of positive relationship between the total protein content of serum and the protein synthesis of body tissues; as the total protein content of serum increases, the effect of tissue protein synthesis will become stronger, and its effect on promoting the growth of tissue organs will also become stronger (25). The addition of brown rice from storage japonica in this assay produced a significant increase in the total protein content in the serum of the pigs used, which may be explained by the higher crude protein content of brown rice from storage japonica than from maize. Total protein can reflect both the level of nutrient metabolism of the animal body, the body’s immune function, and the health of the liver (26) because the liver is the primary site where blood protein is produced, and if total serum protein is significantly reduced, it indicates liver damage (27). This indicates that adding brown rice from storage japonica has no adverse effect on pig health among those in the fattening stage. The serum urea nitrogen level is an indicator that can reflect the body’s protein metabolism and nutritional status (28). The decrease in urea nitrogen content in serum indicates a weakened decomposition of amino acids in animals, an increase in nitrogen storage, and a strengthened protein synthesis effect (29). When the metabolic status of proteins and amino acids changes, the concentration of serum urea nitrogen also changes, and the imbalance of amino acids leads to the degradation of excess amino acids through oxidation, increasing the rate of urea synthesis and resulting in an increase in plasma urea nitrogen levels. Generally, the lower the amino acid utilization rate, the higher the serum urea nitrogen concentration. Similarly, studies have found a significant negative correlation between the growth of lean tissue and the concentration of serum urea nitrogen (30). Therefore, the urea nitrogen content in serum can reflect the animal’s utilization of protein and amino acids in feed. The results of this experiment showed that compared with the CON group, the addition of storage japonica rice brown rice significantly reduced the serum urea nitrogen content of Min pigs, indicating that during the fattening period, Min pigs improved the utilization of amino acids in storage japonica rice brown rice feed and enhanced protein synthesis. Bile acids can directly or indirectly regulate the gut microbiota composition by activating innate immune genes in the intestine (31). Studies have found that some gut microbiota involve the conversion of primary to secondary bile acids and the production of short-chain fatty acids (32).

The present study showed that SJBA did not change carcass characteristics such as carcass weight and length, pH, drip loss, cooking loss, or shear force compared to those in the corn group but increased the eye muscle area of the SJBR group. Similar to the results of this study, pig diets supplemented with brown rice did not alter carcass characteristics compared to pigs without brown rice but showed some changes in the meat composition of fattening pigs (33). Additionally, the present study also found that bile acids reduced the back fat thickness of Min pigs, although it did not completely improve the carcass characteristics. Bile acids can affect the host’s digestion, absorption, and metabolism of lipids by altering the composition and structure of the gut microbiota (34), which may be due to the effects of metabolites of the gut microbiota (such as SCFAs, secondary bile acids, and trimethylamine) and bacterial factors (such as lipopolysaccharides) (35). Ge et al. found that adding bile acids to AA broiler feed significantly reduced the abdominal fat rate (18). Adding different concentrations of bile acids (0, 75, 150, and 300 mg/kg) to the high-fat diet of juvenile fish can improve their digestion and utilization ability, reduce fat deposition, and improve their meat quality (36). The addition of bile acids to the diet significantly reduced the fat content in the whole body, muscles, and the liver of tilapia, promoting its lipid metabolism (37).

Muscle fibers are the basic unit of muscle tissue, and their type and composition play a decisive role in the growth and development of livestock and poultry, as well as meat quality after slaughter (38). According to different enzyme catalysis (adenosine triphosphate enzyme and succinate dehydrogenase), it can be divided into type I oxidized muscle fibers and type II enzymolysis muscle fibers. According to the diversity of myosin heavy chain (MyHC) isomers, they can be divided into the slow oxidation type (type I), rapid oxidation type (type IIa), intermediate type (type IIx), and rapid glycation type (type IIb) (39). The conversion between mature MyHC isomers follows a certain pattern, and the four types are generally classified as follows: I↔IIa↔IIx↔IIb (40). There are reports that the content of type I, IIa, and IIx muscle fibers in livestock muscles is positively correlated with muscle tenderness, color, and fat content, while the content of type IIb muscle fibers is negatively correlated with meat quality traits (41). The results of this experiment showed that there was no significant effect on the mRNA expression of MyHC IIa, MyHC IIx, and MyHC IIb genes among the groups. After adding bile acid, the mRNA expression level of the MyHC I gene in the LD muscle of growing and fattening pigs significantly increased, indicating that bile acid has a certain positive effect on improving pork quality.

The intestinal microbiota has an important impact on the health of the host. In studies of the gut microbiota related to the growth performance of pigs, the feed is an important influence in modifying the structure of the gut microbiota (42). The level of dietary protein, fiber, and fat can all affect the composition of the pig’s intestinal microbiota, thereby affecting the growth, feed utilization, fat deposition, and inflammatory immunity in pigs (43). It has been shown that feeding brown rice flour completely instead of corn to weaned piglets contributes to an increase in beneficial intestinal bacteria (e.g., *Bifidobacteria* and *Lactobacillus*) and a decrease in serum TGF-β1 concentration (44), suggesting that replacing corn with brown rice can improve intestinal health and enhance immune function. In this study, the Shannon diversity index decreased significantly in the SJBR + BA group, while there was no significant difference in the Chao1 and Simpson index among the three treatments, indicating supplementation of bile acids in dietary SJBR rather reduced the microbial richness compared to individual SJBR. This result may seem incomprehensible; however, a previous study reported that the reduced Shannon index may be related to the expected inhibition of dietary lipid absorption (45). The experimental subjects in this study were Chinese fat-type pigs, whose fat deposition is distinctly different from that of lean-type pigs, which may be the reason for the opposing effects. It needs to be recognized that the mechanism of action needs to be further explored and studied in depth. During the growth period, there were no significant differences in fecal microbial composition at the phylum level between treatment groups; during the fattening period, the relative abundance of phylum *Bacteroidetes* and *Firmicutes* was higher, and genera *Lactobacillus* and *Streptococcus* were lower in the brown rice complete replacement diet group compared to the control group (3), suggesting that the long-term feeding of brown rice affects the intestinal microflora of pigs. Both *Bacteroidea* and *Firmicutes* are obesity-related bacteria (46). In fatter pigs, *Firmicutes* has a higher relative abundance, while *Bacteroidea* has a lower relative abundance (47, 48). This indicates that the pigs in this experiment are overweight, possibly related to insufficient exercise within the limit bar. It is worth noting that after adding storage japonica brown rice, the abundance of *Firmicutes* decreased slightly, but the difference was insignificant. Some studies have reported the correlation between gut microbiota composition and carcass characteristics in pigs and found that the abundance of *Lactobacilli* in the gut microbiota of high-quality pork is higher than that of low-quality pork (49, 50).

Intestinal health depends on the three major barriers of the intestine, namely, the intestinal mucosal barrier, the intestinal immune barrier, and the intestinal biological barrier (51). The effect of *Lactobacillus* on the intestinal mucosal barrier function may be mediated by different bacterial structures and chemical components (52). Soluble proteins isolated from *Lactobacillus rhamnosus* can improve *in vitro* intestinal epithelial barrier disruption by inducing the redistribution of Occludin and ZO-1 proteins (53). *Lactobacillus* is the core microorganism in the pig intestine. It was found that *Lactobacillus* stimulates the expression of cellular immune factors, such as CD4 and TNF-α, and inhibits the NF-κB pathway through metabolically produced active substances, thus promoting the development and maturation of the intestinal mucosal immune system and balancing the intestinal immune response (54). Some studies have shown that 100% replacement of corn with brown rice noodles can promote the increase in beneficial bacteria (such as *Bifidobacteria* and *Lactobacilli*) in the intestine of piglets and can also reduce TGF in the serum-β1 concentration, indicating that replacing corn with brown rice can promote intestinal health and enhance the body’s immunity (44).

Compared with the CON group, adding storage japonica brown rice in this experiment increased the relative abundance of *Lactobacilli*, indicating that SJBR may have a certain positive effect on pork quality and intestinal health during the fattening period. Together with *Spirillum*, it changes primary bile acid into secondary bile acid and SCFAs. Research has shown that the *Ruminococcaceae* genus is an important fiber-degrading bacterium that can degrade fiber substances (55). In this experiment, the addition of SJBR significantly reduced the proportion of *UCG-005* genera in the intestines of pigs, which may be related to the lower crude fiber content of SJBR. The large amount of lactic acid it produces makes it a powerful broad-spectrum bactericide, killing viruses, and acting as an immune modulator. It can inhibit the growth of pathogenic bacteria and can be used as a probiotic to restore the structure of the bacterial community (56). In this experiment, the addition of SJBR increased the proportion of *Lactobacilli* in the intestines of pigs compared to the CON group, which indicated that storage japonica brown rice is beneficial for maintaining intestinal homeostasis in pigs. In contrast, bile acid cannot significantly increase the proportion of probiotics in the intestines of pigs.

## 5 Conclusion

In summary, this study revealed that supplementation of 25% storage japonica brown rice in the feed during the fattening period might have a certain positive effect on the growth performance, meat quality, and intestinal health of growing–finishing Min pigs. Meanwhile, this study found that adding 0.025% bile acid (hyodeoxycholic acid) to 25% storage japonica brown rice feed can significantly reduce back fat thickness and improve meat quality. The current study indicates that replacing part corn with storage japonica brown rice is feasible, but how to further improve the growth efficiency of pigs with the storage brown rice diet is still a key challenge that needs to be studied.

## Data availability statement

The original contributions presented in the study are publicly available. This data can be found at: https://www.ncbi.nlm.nih.gov/bioproject/; PRJNA1076370.

## Ethics statement

The animal study was approved by the Institutional Animal Care and Use Committee of Jilin University (SY202209301). The study was conducted in accordance with the local legislation and institutional requirements.

## Author contributions

CW: Writing – original draft, Formal analysis. KZ: Writing – original draft, Formal analysis. DW: Writing – original draft, Methodology. HY: Writing – original draft, Data curation. YZ: Writing – review & editing, Resources, Data curation. HF: Writing – review & editing, Investigation. JZ: Writing – review & editing, Conceptualization, Funding acquisition.
